# A rapid literature review of the impact of penicillin allergy on antibiotic resistance

**DOI:** 10.1093/jacamr/dlaf002

**Published:** 2025-01-21

**Authors:** Shadia Ahmed, Jonathan A T Sandoe

**Affiliations:** Department of Microbiology, Leeds Teaching Hospitals NHS Trust, Leeds, UK; Leeds Institute of Medical Research, School of Medicine, University of Leeds, Leeds, UK; Department of Microbiology, Leeds Teaching Hospitals NHS Trust, Leeds, UK; Leeds Institute of Medical Research, School of Medicine, University of Leeds, Leeds, UK

## Abstract

**Background:**

Antimicrobial resistance (AMR) is caused by the use and misuse of antibiotics. AMR is a global health concern, to which penicillin allergy (penA) labels appear to contribute. Patients who have penA labels are treated with non-penicillin antibiotics and receive more antibiotics when compared with patients without penA. Although penA is common, after formal allergy assessments, >90% of people with a penA label are found not to be allergic; therefore, broad-spectrum antibiotics are being misused and overused unnecessarily in these patients. Antimicrobial stewardship policies now advocate assessment to identify and remove incorrect penA labels; however, there is limited evidence on whether rectifying incorrect penA labels results in less AMR. This review aimed to assess the association between AMR and antibiotic allergy labels.

**Methods:**

A comprehensive literature search using EMBASE and MEDLINE databases was conducted. Studies were included if they compared the presence of infection or colonization with an antimicrobial-resistant organism in participants with and without antibiotic allergy.

**Results:**

Thirty-three studies were included, and all the studies were observational in design and included a variety of patient groups. Eighteen studies compared AMR outcomes in participants with and without penA, and the rest investigated the impact of beta-lactam allergy or any antibiotic allergy on AMR outcomes. MRSA was the most investigated pathogen, and 11 of 13 studies showed that penA was associated with MRSA. PenA labels were also associated with vancomycin-resistant enterococci (three of five studies). There was limited evidence on the impact of penA on extended-spectrum beta-lactamase-producing Enterobacterales and resistant *Streptococcus pneumoniae*.

**Conclusion:**

The presence of penA labels is associated with antibiotic resistance in key pathogens in a wide variety of patient groups.

## Introduction

Incorrect penicillin allergy (penA) labels have become an important barrier to the safe treatment of infection and affect an estimated 2.7 million people in England.^1^ In the UK, 6% of primary care and 14% of hospitalized patients are reported to have penA, but only a fraction of these patients have a confirmed allergy on formal testing.^1–4^ PenA is associated with higher rates of treatment failure, higher mortality, *Clostridioides difficile* infection and increased treatment costs but also affects antibiotic exposure.^1,5–8^ Patients with penA labels are more likely to receive broad-spectrum second-line antibiotics, such as carbapenems, tetracyclines, quinolones and macrolides,^1,9–11^ and are less likely to have their antibiotic regimen narrowed.^10^

The ‘AWaRe’ antibiotic classification tool developed by the WHO^12^ classifies antibiotics into three groups to guide their optimal use based in part on their potential for causing antimicrobial resistance (AMR): Access (first- or second-line antibiotics with a lower propensity to drive AMR), Reserve (antibiotics with a higher propensity for AMR) and Watch (last-resort antibiotics with the highest concern about AMR). A UK cohort study found that patients with penA are more likely to be prescribed non-access group antibiotics, and thus, it is plausible that the presence of a penA label could contribute to the emergence and selection of AMR.^13^

**Figure 1.**
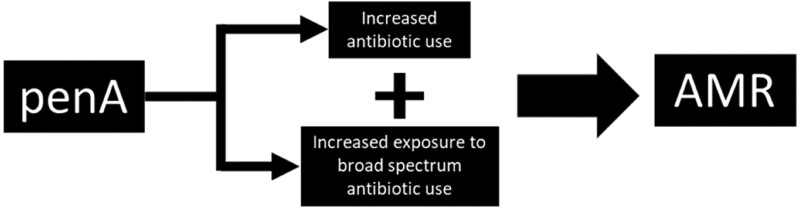
Visual representation of the hypothesized pathway between penA and AMR.

As well as being treated with broad-spectrum agents, overall antibiotic use is higher in penA patients. In primary care, studies have shown that penA patients receive more antibiotic prescriptions compared with those without a penA.^1,9^ Exposure to ‘broad-spectrum’ antibiotics as well as increased antibiotic use in patients with penA is likely to predispose to AMR infections (Figure 1). Over 90% of patients with a penA label are able to tolerate penicillins when formally tested;^4^ therefore, for a significant proportion of patients, this label can be removed and antibiotic use optimized. As such, allergy testing to identify and remove invalid penA labels (‘de-labelling’) has been suggested as an antimicrobial stewardship strategy to tackle AMR;^14^ however, the impact de-labelling has on AMR is largely unknown. This review explores the association between penA and other antibiotic allergies and AMR, as well as the effect of de-labelling penA on AMR.

## Methods

**Figure 2.**
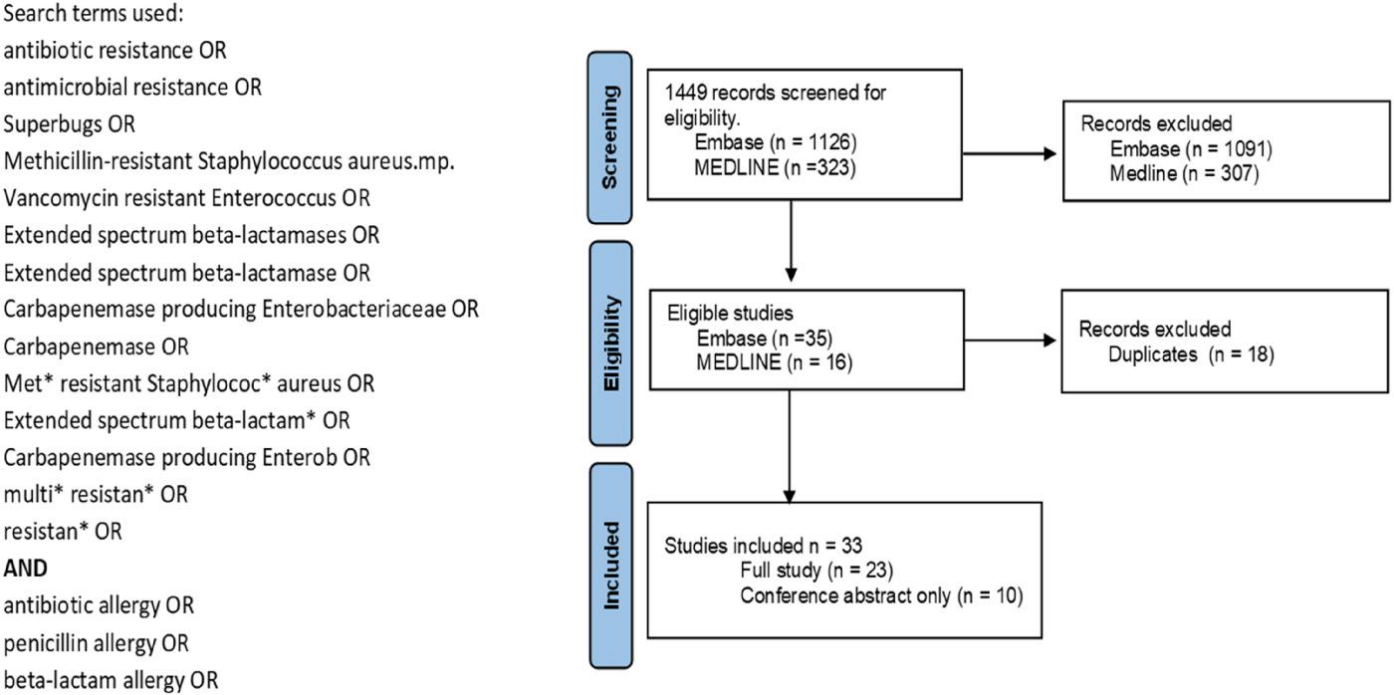
Search terms and flow diagram outlining identification of studies investigating the impact of penA on AMR.

A literature search of EMBASE and MEDLINE databases was conducted to identify published studies that fit the PECOS parameters defined in Table 1, and the search was carried out in October 2023 and repeated in June 2024. This review, although focused on the impact of penA on AMR, also considered the impact of other antibiotic allergies on AMR. To be included, studies had to compare AMR outcomes in participants with and without antibiotic allergy. Only articles in the English language were included. Search terms and flow diagram of the screening process are shown in Figure 2.

**Table 1.** PECOS (population, exposure, comparator, outcome, study design) parameters

Population	Adult and paediatric patients in community and hospitals
Exposure	Patients with an antibiotic allergy or beta-lactam allergy or penA who may or may not have undergone penA de-labelling
Comparator	Patients without the exposure
Outcome	AMR outcomes defined as either infection or colonization with an antimicrobial-resistant organism.
Study design	Observational or interventional studies. Review articles not included (including systematic reviews)

## Results

Titles and abstracts of 1449 articles were screened before a full-text review of articles that met the inclusion criteria (Figure 2).

Thirty-three studies fulfilled the inclusion criteria and were included (Table S1, available as Supplementary data at *JAC-AMR* Online). Nine were conference abstracts only.^15–23^ Eight studies^17,24–30^ investigated the impact of beta-lactam allergy on AMR outcomes. Five^18,31–34^ looked at the impact of any antibiotic allergy on AMR outcomes. Eighteen studies^1,3,15,16,19–23,35–42^ compared AMR outcomes in patients with and without penA, and two^43,44^ specifically compared AMR outcomes in participants with and without penicillin and/or cephalosporin allergies.

### Methicillin-resistant *Staphylococcus aureus*

**Figure 3.**
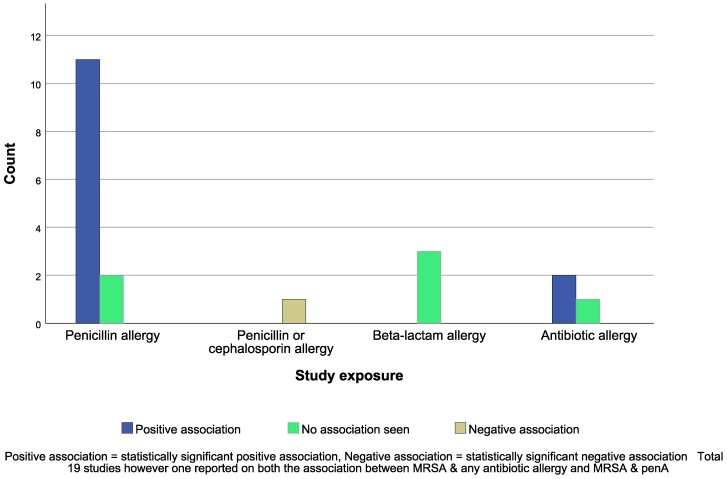
Studies reporting the association between antibiotic allergy labels and MRSA grouped by study exposure.

In total, 19 studies investigated the link between MRSA and antibiotic allergy (Table 2). The majority of these (58%) were set in the USA. Eleven (85%) of the 13 studies with MRSA as an outcome found an association between patients with penA labels and an increased risk of MRSA (Figure 3).^1,3,15,20–22,35,36,39,41,45^

**Table 2.** Studies reporting the association between allergy labels and MRSA

Authors	Country	Patient group	Exposure	Study controlled for confounding	Association with MRSA
Reddy et al.^15^	USA	Inpatients	Penicillin allergy	No	Positive
Macy et al.^39^	USA	Inpatients	Penicillin allergy	Yes^a^	Positive
Tan et al.^44^	USA	Surgical	Penicillin or cephalosporin allergy	Yes^b^	Negative
Jones et al.^18^	USA	CF	Antibiotic allergy	No	Positive^o^
Sousa-Pinto et al.^35^	Portugal	Inpatients	Penicillin allergy	Yes^c^	Positive
Blumenthal et al.^36^	UK	Primary care	Penicillin allergy	Yes^d,e^	Positive
Galant-Swafford et al.^20^	USA	Inpatients	Penicillin allergy	Yes^f^	Positive^o^
West et al.^1^	UK	Primary care	Penicillin allergy	Yes^g,h^	Positive
Strazzulla et al.^27^	France	ICU	Beta-lactam allergy	No	No association seen
Motoa et al.^25^	USA	SOT	Beta-lactam allergy	Yes^i^	No association seen
Lam et al.^33^	USA	Inpatients	Antibiotic allergy	No	Positive
			Penicillin allergy^m^		No association seen
Baxter et al.^3^	UK	Inpatients	Penicillin allergy	No	Positive
Schlosser et al.^45^	USA	Surgical	Penicillin allergy^n^	No	Positive
Nelson et al.^42^	USA	SOT	Penicillin allergy	No	No association seen
Greenwald et al.^22^	USA	CD/UC	Penicillin allergy	Yes^j^	Positive
Chakravorty et al.^31^	Australia	Inpatients	Antibiotic allergy	Yes^k^	No association seen
Strazzulla et al.^28^	France	ICU	Beta-lactam allergy	No	No association seen
Greenwald et al.^21^	USA	CD/UC	Penicillin allergy	Yes^j^	Positive
Jones et al.^41^	UK	Surgical	Penicillin allergy	Yes^l^	Positive

Positive = statistically significant positive association; Negative = statistically significant negative association.

Abbreviations: BMI, body mass index; CCI, Charlson comorbidity index; CD, Crohn’s disease; CF, Cystic fibrosis; ICU, intensive care unit; IMD, index multiple deprivation; PPI, proton pump inhibitor; SOT, solid organ transplant; UC, ulcerative colitis.

^a^Patients matched on primary diagnosis, sex, age and admission date.

^b^Logistic regression modelling which controlled for age, BMI, institutional site, gender, joint, year of surgery and CCI.

^c^Patients matched on age, sex and main diagnosis.

^d^Patients matched on age, sex and study entry time.

^e^Cox proportional hazard modelling which controlled for age, sex, BMI, socioeconomic status, smoking status, alcohol use, CCI, haemodialysis, number of antibiotic prescriptions, PPI use, corticosteroid use, other antibiotic allergies, resident of nursing home, visits to a GP and admissions to hospital.

^f^Authors state confounders controlled for but details not provided.

^g^Patients matched on age, sex, ethnicity, IMD, comorbidities and the proportion of patients with a penA record within the general practice.

^h^Binomial modelling adjusted for age, sex, ethnicity, IMD, smoking status, comorbidities and the proportion of patients with a penA record within the general practice.

^i^Propensity score matching with logistic regression, controlled for liver diagnosis groups, intraoperative continuous renal replacement therapy, year of transplant, age at diagnosis, donor risk index, operative time, blood loss, raw model of end-stage liver disease and donation after circulatory death.

^j^Matched on age, sex, race, glucocorticoid use and PPI use.

^k^Logistic regression, which controlled for age, sex, immunocompromised status and medical specialty, reported outcome incident MRSA + *C difficile* infection.

^l^Logistic regression modelling which controlled for BMI, receipt of inpatient antimicrobials in the 6 months prior to surgery and length of hospital stay.

^m^Subgroup analysis.

^n^Included 1.8% patients with other β-lactam allergy.

^o^Significance results not reported.

Two large studies that controlled for potential confounders reported on MRSA in primary care patients.^1,36^ A population-based cohort study utilizing the UK Health Improvement Network electronic health record (EHR) database^36^ followed up patients without a prior history of MRSA. Patients with penA were found to have a 69% higher risk of MRSA colonization and/or infection (demonstrated by the presence of clinical codes indicating either MRSA infection, carriage, eradication or decontamination) compared with those without (adjusted HR 1.69, 95% CI 1.51–1.90).^36^ Further evaluation in this study found that this increased risk was mediated by the use of macrolides, clindamycin and fluoroquinolones, whereas the use of penicillin was not associated with increased MRSA risk. Another large study utilizing UK primary care health records (Research One) and an exact matching methodology found penA was associated with a higher prevalence of MRSA infection/colonization (RR 1.90, 95% CI 1.50–2.41).^1^

MRSA was also found to be associated with penA in secondary care patients.^3,15,20–22,35,39,41,45^ In a point prevalence study of penA in inpatients of a UK National Health Service hospital, increased rates of MRSA were seen in patients with penA compared with those without (10.3% versus 2.23%, *P* = 0.0065).^3^ Similarly, a large multicentre retrospective cohort study of patients hospitalized in Portuguese public hospitals, which used data from a national database, found patients with penA had higher rates of MRSA (0.3% versus 0.2%, *P* < 0.001) infections.^35^

In a case–control study using data collected from EHR of hospitalized patients admitted to a single centre in the USA,^39^ penA patients were found to have a higher prevalence of MRSA (OR 1.14, 95% CI 1.07–1.32).^39^ Similarly, MRSA infection was significantly higher in penA patients compared with controls (0.69% versus 0.21%) in a cohort of inpatients admitted to a US university hospital.^20^ The authors stated that confounders were controlled, however only a conference abstract was available, and details of confounders were not reported. In another retrospective USA-based study that utilized MRSA surveillance data captured in EHRs, patients who had MRSA were more likely to have penA than the general hospital population (12% versus 6%, *P* < 0.05).^15^ Prevalence of MRSA was also higher in patients with other antibiotic allergies not including penA.

Patients with penA undergoing abdominal surgery have also been found to have an increased risk of MRSA.^41,45^ In a retrospective review of a prospective, institutional, hernia-specific database in the USA, patients undergoing open ventral hernia repair who had a penA were more likely to have a history of MRSA at presentation (11.0% versus 6.3%, *P* = 0.01).^45^ In a UK-based cohort of patients undergoing gastrointestinal surgery, pre-operative MRSA infection/colonization was more prevalent in patients with penA (1.7% versus 0.8%, *P* = 0.027).^41^ PenA patients also had a higher risk of acquiring new MRSA infection/colonization post-operatively within 60 days when adjusted for BMI, receipt of in-hospital antimicrobials in the 6 months prior to surgery and length of hospital stay [adjusted OR (aOR) 2.82, 95% CI 1.18–6.75].^41^ Additionally, when a beta-lactam was utilized in the surgical prophylaxis regimen, the odds of MRSA acquisition were reduced compared with regimens containing a backbone of either ciprofloxacin or gentamicin with metronidazole, although this was not found to be significant in the multivariable analysis (aOR 0.50, 95% CI 0.20–1.27).^41^ It is possible that the increased risk of MRSA seen in the non-β-lactam-containing prophylaxis regimen was driven by the use of ciprofloxacin which has been associated with the isolation of MRSA.^46–48^

Two studies investigated the impact of penA on MRSA in patients with inflammatory bowel disease.^21,22^ One study included patients with either ulcerative colitis (UC) and Crohn’s disease (CD) and utilized propensity score matching of patients with and without penA: patients with UC and CD had a higher risk of MRSA infection (OR 1.81, 95% CI 1.34–2.46, *P* < 0.0001, and 1.76, 95% CI 1.38–2.2, *P* < 0.0001, respectively).^22^ Another matched controlled study that just included patients with UC also found patients with UC had an increased risk of MRSA infection (OR 1.73, *P* < 0.001).^21^ Conversely, in a retrospective study set in the USA that investigated the impact penA labels had on organ transplant patients admitted with a primary infectious process, no difference in rates of MRSA infection was seen in patients with and without a penA (1.6% versus 1.6%; *P* = 0.92).^42^ This study used ICD-9 codes to identify patients with penA and infection outcomes which may affect study validity.

Three studies reported on the association between beta-lactam allergy and MRSA.^25,27,28^ In patients admitted to French ICUs, patients with beta-lactam allergies were not found to have an increased risk of MRSA colonization or acquisition.^27,28^ Similarly, in US liver transplant recipients, beta-lactam allergy was not associated with MRSA colonization (2/87 [2.3%] versus 3/174 [1.7%], *P* = 0.75) and infection (1/87 [1.1%] versus 3/174 [1.7%], *P* = 0.72).^25^

A two-centre retrospective study based in the USA investigated the impact vancomycin prophylaxis had on penA- or cephalosporin-allergic patients undergoing joint arthroplasty.^44^ In this study, penicillin- or cephalosporin-allergic patients who received vancomycin prophylaxis for their surgery were compared with non-allergic patients who received cefazolin. Results showed more favourable outcomes in patients receiving vancomycin, who had a reduced risk of prosthetic joint infections (PJI) with antibiotic-resistant organisms (aOR 0.10, 95% CI 0.01–0.88, *P* = 0.038), defined as isolation of either MRSA or VRE, but there were no cases of VRE in the study.^44^

Three studies looked at the impact of any antibiotic allergy on MRSA, and two (67%) found a positive association.^18,33^ A single-centre study set in the USA found cystic fibrosis (CF) patients with any antibiotic allergy had a 2-fold higher risk of MRSA cultured from sputum when compared with non-allergic CF patients.^18^ Additionally, a US single-centre study of adult inpatients undergoing surveillance screening for MRSA and VRE^33^ reported patients with any antibiotic allergy were more likely to be colonized with MRSA (8.3% versus 4.7%, *P* = 0.025). In a subgroup analysis of specific antibiotic allergy labels, there was no difference in MRSA colonization in penA and non-penA patients, while tetracycline and glycopeptide allergies were associated with higher rates of MRSA colonization. Another study reported no difference in the incidence of MRSA in patients with any antibiotic allergy versus those without, in a small cohort of patients referred for antimicrobial stewardship review and assessment.^31^ This cohort was subject to selection bias as it only included inpatients identified by pharmacists during ward rounds.

### Vancomycin-resistant enterococci

In total, six studies reported on VRE,^3,15,16,22,39,41^ five reported on the association between penA and VRE, while one study reported on the association between VRE and any antibiotic allergy^33^ (Table 3). Two studies set in the USA found that penA prevalence was higher in patients with VRE compared with the general hospital population (24–28% versus 6–8%).^15,16^ This association was also observed in patients with other antibiotic allergies not including penA.^15^ Patients with penA were also more likely to receive vancomycin.^16^ In another EHR-based case–control study of hospitalized patients in the USA, penA case patients were found to have more VRE infections when compared with non-penA control patients (OR 1.30, 95% CI 1.13–1.50).^39^ Case patients were more likely to receive clindamycin, fluoroquinolones, vancomycin, and third-generation cephalosporins.^39^

**Table 3.** Studies reporting the association between allergy labels and VRE

Authors	Country	Patient group	Exposure	Study controlled for confounders	Association with VRE
Reddy et al.^15^	USA	Inpatients	Penicillin allergy	No	Positive
Macy et al.^39^	USA	Inpatients	Penicillin allergy	Yes^a^	Positive
Reddy et al.^16^	USA	Inpatients	Penicillin allergy	No	Positive^c^
Lam et al.^33^	USA	Inpatients	Antibiotic allergy	No	No association
Baxter et al.^3^	UK	Inpatients	Penicillin allergy	No	No association
Jones et al.^41^	UK	Surgical	Penicillin allergy	Yes^b^	No association

Positive = statistically significant positive association; Negative = statistically significant negative association.

^a^Patients matched on primary diagnosis, sex, age and admission date.

^b^Logistic regression modelling which controlled for BMI, receipt of in hospital antimicrobials in the 6 months prior to surgery and length of hospital stay.

^c^Significance results not reported.

Conversely, post-operative VRE infection/colonization was not associated with penA in patients who had undergone gastrointestinal surgery (aOR 1.10, 95% CI 0.51–2.37, *P* = 0.814).^41^ In a point prevalence study conducted in a UK-based hospital, higher rates of VRE in hospitalized penA patients were seen; however, this did not reach statistical significance (3.8% versus 1.41%, *P* = 0.09).^3^

Additionally, in a study investigating the association between antibiotic allergies and VRE, there was no significant difference in VRE colonization in patients with and without any antibiotic allergies (36.2% versus 46.9%, *P* = 0.10).^33^

### Other Gram-positive bacteria

There are some data that suggest the presence of any antibiotic allergy is associated with increased macrolide resistance in *Streptococcus pneumoniae*.^19,34^ One US study assessed the impact of the presence of any antibiotic allergy on resistance in *S. pneumoniae*.^34^ This retrospective cohort study investigated risk factors associated with resistance in patients treated for pneumococcal bacteraemia (*n* = 1574) and found that having an antibiotic allergy was an independent risk factor (OR 1.7, 95% CI 1.1–2.6) for macrolide resistance^35^ when adjustment was made for age, sex, race, prior antibiotic use, immunosuppression, region of residence within the USA, and type of healthcare insurance. There were no significant differences seen for penicillin or cephalosporin resistance (OR 1.3, 95% CI 0.9–1.8 and OR 1.4, 95% CI 0.9–2.3, respectively). In a UK-based study, a comparison was made between resistance profiles in clinical isolates from penA and non-penA patients.^19^ There was no significant difference between penA and non-penA patients in resistance profiles of blood culture specimens isolating *S. aureus* and *S. pneumoniae*; however, a higher proportion of patients with penA had resistance in *S. pneumoniae* sputum isolates to macrolides and doxycycline compared with those without (12.6% versus 20.7% and 18.9% versus 24.1%, respectively), although this finding was not statistically significant. This trend is concerning as patients with penA will often be treated with macrolides or doxycycline in place of penicillin. This raises concerns about the potential risk of resistance to alternative therapies, which is associated with the presence of the penA label.

In a recent retrospective study, patients with either a penicillin or cephalosporin allergy who received non-cefuroxime, second-line prophylaxis (>97% received clindamycin) for hip or knee arthroplasty were compared with patients without allergy who received cefuroxime prophylaxis.^43^ The susceptibility profiles of causative bacteria in subsequent PJI were investigated. Coagulase-negative staphylococci (CNS) were the most commonly isolated pathogens. Patients with either penicillin or cephalosporin allergies who received non-cefuroxime prophylaxis for hip arthroplasties were more likely to have tetracycline-resistant CNS (2/23, 8.7% versus 3/3 100%, *P* < 0.001). No significant differences for the other antibiotics were seen. Additionally, when tested, increased resistance to oxacillin (20/48 isolates versus 3/3 isolates, *P* = 0.049), ciprofloxacin (15/58 versus 3/3, *P* = 0.006), moxifloxacin (16/58 versus 3/4 *P* = 0.047) and tetracycline (2/39 versus 4/4, *P* < 0.001) was seen in penicillin-/cephalosporin-allergic patients who received non-cefuroxime surgical prophylaxis. In cases of PJI post-total/unicompartmental knee arthroplasty, allergic patients who received non-cefuroxime prophylaxis were more likely to have fosfomycin-resistant CNS (2/10 20% versus 2/2 100%, *P* = 0.03) but no significant differences were found for the other agents tested. Resistance to co-trimoxazole (1/16 isolates versus 2/3 isolates, *P* = 0.009), fosfomycin (2/19 versus 2/2, *P* = 0.002) and linezolid (0/19 versus 2/2, *P* < 0.001) was more prevalent in the non-cefuroxime cohort who had PJI post-total/unicompartmental knee arthroplasty. However, the overall numbers in each category when comparing resistance in allergic and non-allergic cohorts are small.

### Resistant Gram-negative bacteria

Studies reporting on the impact of antibiotic allergies and resistance in Gram-negative organisms were heterogeneous in their study populations and outcomes (Table 4). A UK-based cohort study looking at the impact of penA labels on surgical site infections found that penA patients had lower odds of acquiring post-operative infection/colonization with Gram-negative bacteria resistant to third-generation cephalosporins in both the univariable and multivariable analyses (aOR 0.37, 95% CI 0.16–0.87).^41^ This finding may be explained by the fact that patients with penA were less likely to have had a β-lactam-based antibiotic prophylactic regimen and had either a ciprofloxacin- or gentamicin-based regimen instead (*P* = 0.001).

**Table 4.** Studies reporting the association between allergy labels and gram-negative bacteria

Authors	Country	Patient group	Exposure	Study controlled for confounding	Association with resistance in Gram-negative bacteria
Jones et al.^18^	USA	Cystic fibrosis	Antibiotic allergy	No	Mixed^f^
Strazzulla et al.^27^	France	ICU	Beta-lactam allergy	Yes^a^	Positive^g^
Motoa et al.^25^	USA	SOT	Beta-lactam allergy	Yes^b^	No association seen^g^
Baxter et al.^3^	UK	Inpatients	Penicillin allergy	No	No association seen^g,h^
Ahmed et al.^19^	UK	Mixed	Penicillin allergy	No	No association seen^i^
Strazzulla et al.^28^	France	ICU	Beta-lactam allergy	Yes^c^	No association seen in multivariate analysis^g^
Naciri et al.^26^	France	Surgical	Beta-lactam allergy	Yes^d^	No association seen^j^
Jones et al.^41^	UK	Surgical	Penicillin allergy	Yes^e^	Negative^j^

Positive = statistically significant positive association; Negative = statistically significant negative association.

Abbreviations: ICU, intensive care unit; SOT, solid organ transplant

^a^Logistic regression modelling that used results from univariate analysis, model included ESBL organisms at admission, sex and Simplified Acute Physiology Score (SAPS) II.

^b^Propensity matching included sex, liver diagnosis groups, intraoperative continuous renal replacement therapy, year of transplant, age at diagnosis, donor risk index, operative time, blood loss, raw model of end-stage liver disease and donation after circulatory death.

^c^Logistic regression modelling that used results from univariate analysis, model included ESBL organisms at admission, ESBL organisms at discharge and sex.

^d^Patients matched on age, sex, disease severity, site of infection and whether infection was healthcare-associated.

^e^Logistic regression modelling which included duration of procedure, receipt of in hospital antimicrobials in the 6 months prior to surgery, number of antimicrobial doses in the 28 days after surgery and length of hospital stay.

^f^Negative association with ESBL-producing *Klebsiella oxytoca* and positive association with *S. maltophilia* and *A. xylosoxidans.*

^g^Extended-spectrum beta-lactamase (ESBL)-producing Gram-negative bacteria.

^h^AmpC-producing Gram-negative bacteria.

^i^ *Haemophilus influenzae.*

^j^Fluoroquinolone or third-generation cephalosporin-resistant Enterobacterales.

The impact of beta-lactam allergy on patients with intra-abdominal infections (IAI) has also been recently investigated in a small retrospective case–control study.^26^ In this study, patients with a beta-lactam allergy treated with either a fluoroquinolone or aztreonam plus metronidazole were matched with controls treated with beta-lactam antibiotics (not including aztreonam). Patients without a beta-lactam allergy were more likely to isolate fluoroquinolone or third-generation cephalosporin-resistant Enterobacterales from either surgical samples or blood cultures (6/23, [26%] versus 2/17 [12%]), although this was not reported to be statistically significant. In this study, ‘cases’ included patients with a beta-lactam allergy as well as patients treated as such based on the antibiotics they received for their IAI; therefore, the sample may not be truly representative of patients with beta-lactam allergies.

A UK-based study of patients who had *H. influenzae* cultured from sputum found no significant difference in resistance profiles of the *H. influenzae* isolates when comparing patients with and without a penA.^19^ Five studies reported the impact of antibiotic allergies on extended-spectrum beta-lactamase (ESBL)-producing Gram-negative bacteria.^3,18,25,27,28^ Patients admitted to an ICU who had beta-lactam allergies had higher rates of ESBL carriage at admission (6/45 [13.3%] versus 49/1129 [4.3%], *P* = 0.022) and on discharge (9/45 [20.0%] versus 102/1129 [9.0%], *P* = 0.046) compared with patients without these allergies.^27^ Only ESBL carriage at admission was significantly associated with a beta-lactam allergy in the multivariate analysis (RR 3.00, *P* = 0.0191). This trend was confirmed in a later article reporting the complete study of 3332 ICU patients investigating multidrug-resistant organisms in patients with and without beta-lactam allergies: in this cohort^28^ patients with a beta-lactam allergy had higher rates of ESBL carriage on admission (19/132 [14.4%] versus 248/3200 [7.8%], *P* = 0.01) and at discharge (22/132 [16.7%] versus 352/3200 [11%] *P* = 0.04); however, these were not found to be significant in the multivariate analysis. Details regarding differences in antibiotic exposures were not reported.

In a UK cohort, no differences were seen in rates of Gram-negative bacteria producing AmpC and ESBL between patients with and without penA (7.6% versus 6.67% and 3.8% versus 3.64% respectively).^3^ In a USA-based study of liver transplant recipients with and without a beta-lactam allergy, infection or colonization with ESBL-producing Enterobacterales was not associated with beta-lactam allergies (ESBL infection 2/87 [2.3%] versus 5/174 [2.9%], *P* = 0.79 and colonization 2/87 [2.3%] versus 4/174 [2.3%] *P* > 0.09).^25^ A retrospective cohort study of patients with CF found that in sputum samples from antibiotic-allergic patients, ESBL-producing *Klebsiella oxytoca* was cultured more than two times less often when compared with non-allergic patients. This study also found that patients with CF were twice as likely to isolate *Stenotrophomonas melophilia* and *Alcaligenes xylosoxidans*, which are intrinsically resistant to several antibiotic classes.^18^

### Multidrug-resistant bacteria

Twelve studies reported on the presence of MDR bacteria or antibiotic resistance; however, the definition of what constituted an MDR organism/resistance differed between studies.^17,23,24,27–30,32,35,37,40,42^ Seven of the studies (58%) reported no significant association between AMR and allergy labels.^17,27,29,30,32,40,42^ One retrospective matched cohort study of liver transplant recipients found that patients with antibiotic allergies did not have statistically significantly higher rates of MDR Gram-negative organism isolation when compared with patients without an antibiotic allergy (4/51 [8%] versus 1/52 [2%], *P* = 0.20) despite increased use of cephalosporins (30% versus 23%, *P* = 0.03).^32^ This study adopted the Magiorakos definition of MDR (defined as resistance to at least one agent in three or more antibiotic classes).^49^ Similarly, a retrospective study of solid organ transplant (SOT) recipients (liver, kidney, lung, heart, pancreas) found no difference in the incidence of MDR organisms, isolated in clinical samples in the post-transplant period in patients with beta-lactam allergies compared with those without (aOR 1.0, 95% CI 0.7–1.6, *P* = 0.83); this was a multivariate analysis that controlled for transplanted organ, age, sex, diabetes mellitus, pretransplant hospitalization status, receipt of induction therapy and initial antimetabolite drug. MDR was defined as isolation of MRSA, VRE, extended-spectrum cephalosporin-resistant Enterobacterales, carbapenem-resistant Enterobacterales or MDR *Pseudomonas aeruginosa*.^30^ Included patients with β-lactam allergy were more likely to receive aminoglycosides, aztreonam, clindamycin, fluoroquinolones and vancomycin in the post-transplant period (*P* < 0.001) and less likely to receive cephalosporins or penicillins (*P* < 0.001).

In patients admitted to a French ICU with sepsis, beta-lactam allergy was associated with an increased rate of infection with MDR organisms (3.4% versus 1.9%, *P* < 0.027).^24^ In another French ICU cohort, patients with beta-lactam allergies had higher rates of MDR organism carriage on admission in the univariate analysis (18.2% versus 11.4, *P* = 0.04).^28^ In both these studies, there was no clear definition of which isolates were categorized as MDR.

Two further studies did not provide information on how they measured their AMR outcomes. In a US electronic healthcare record-based analysis of patients who had undergone insertion of a left ventricular assist device, patients with penA were more likely to be documented as having AMR during a 5-year follow-up period (*P* = 0.009) compared with patients without penA following adjustment for age, gender, race, ethnicity and diagnosis of hypertension and heart failure.^23^ Conversely, there were no significant differences in the burden of bacterial resistance seen in patients with and without a beta-lactam allergy label who were hospitalized in an Australian tertiary hospital.^17^ Both studies were only available as conference abstracts, lacking further information regarding design and methodology.

Two studies used ICD-9 codes to identify MDR organisms: a large retrospective study of patients admitted to Portuguese hospitals found higher rates of drug-resistant infections in patients with penA (0.11% versus 0.08, *P* 0.02)^35^ and in a subgroup analysis of medical and surgical admissions, medical inpatients with penA had significantly higher rate of drug-resistant infections. In a USA-based study of SOT recipients admitted with a primary infectious process, no difference was seen in rates of multidrug-resistant organisms observed.^42^ As drug resistance data were obtained from ICD-9 codes, more detailed information regarding specific pathogens is not available.

A 5-year retrospective single-centre study reviewed the impact of penA on AMR in patients treated for complicated odontogenic infection (defined as any odontogenic infection requiring admission and surgical management).^37^ AMR in this study was defined as any cultured bacterial species that demonstrated resistance to either clindamycin, penicillin or levofloxacin, and included both complete and intermediate resistance. Details on sample type and isolated organisms are not provided; however, patients with penA were found to have a higher risk of AMR overall (RR 2.34, 95% CI 1.66–3.32, *P* < 0.001), as well as clindamycin resistance (RR 3.17, 95% CI 1.93–5.18, *P* < 0.001). There was also a non-significant trend towards an increased risk of penicillin resistance in penA patients (RR 1.98, 95% CI 0.81–4.83, *P* = 0.13).^37^

### Ciprofloxacin resistance in urinary tract infections

A retrospective cohort study of 6361 US inpatients with community onset urinary tract infections (UTI) found patients with penA were more likely to have a ciprofloxacin-resistant UTI compared with those without (aRR 1.13, 95% CI 1.06–1.19) when adjusted for confounders (age, sex, comorbidities, healthcare exposure, carbapenem allergy and cephalosporin allergy).^38^ Mediation analysis found that this increased risk was partially mediated by the use of fluoroquinolones in the 90 days prior to the UTI. In this study, *Escherichia coli* (*n* = 2797), *Enterococcus faecalis* (*n* = 1281), *Klebsiella pneumoniae* (*n* = 876), *Pseudomonas aeruginosa* (*n* = 391) and *Enterococcus faecium* (*n* = 292) accounted for 75.9% of all isolates.

### Impact of allergy assessment on AMR

Only two studies looked at the impact of antibiotic allergy assessment on AMR.^29,40^ A matched case–control study,^29^ investigating the impact of penA testing in patients with ‘low-risk’ allergy histories on antibiotic use and infection-related outcomes, with 155 patients/arm, showed no significant difference in MDR organism isolation 12 months after testing (OR 1.09, 95% CI 0.48–2.47), despite a significant reduction in restricted antibiotic use (OR 0.48, 95% CI 0.28–0.82), restricted antibiotics included lincosamides, fluoroquinolones, vancomycin, carbapenems and third (and subsequent)-generation cephalosporins. Cases and controls were matched based on age, sex, admitting unit, immune status and number of hospital admissions, and no formal power calculations were performed. MDR organisms were defined as either colonization with MRSA, VRE, MDR Gram-negative organisms (defined as resistance to third-generation cephalosporin or carbapenem) or infection with *Clostridioides* (*Clostridium*) *difficile*. A second study of 56 patients with penA assessed the impact of antibiotic desensitization on patient outcomes.^40^ This retrospective, case–control study included participants with high-risk penA histories (confirmed allergy with positive skin tests or history of immediate hypersensitivity reaction or unconfirmed history but clinically unstable and in need of a penicillin antibiotic). Cases were defined as penA patients with an infection who underwent antibiotic desensitization, and controls were penA patients with an infection managed with an alternative non-penicillin antibiotic. In patients who were readmitted within 30 days, four patients in the control group had new MDR organism acquisition compared with zero patients in the case group; MDR was defined using the Magiorakos definition for MDR organisms.^49^

## Discussion

This review has identified that both penA labels and labels of allergy to any antibiotic have been associated with infection and/or colonization with AMR microorganisms in a wide range of key pathogens, patient groups and settings across the world. In most cases, the effect was towards increased resistance in those with allergy, but this was not always the case. This finding is consistent with a systematic review by Krah et al.^50^ which found that 10 of 15 studies demonstrated patients with antibiotic allergy labels were more likely to be infected/colonized with an MDR organism. While an association has been found, causality between penA labels and AMR cannot be definitively established due to the observational nature of the studies.

Some studies lumped all beta-lactam allergies together, but this may hamper detection of a relationship between allergy and AMR because penA and cephalosporin allergy have different effects on antibiotic prescribing. Lumping all antibiotic allergies together is also methodologically problematic because prescribing behaviour may be pushed in opposite directions depending on allergy status—for example, co-trimoxazole allergy may increase beta-lactam use, while penA increases co-trimoxazole use. This may explain why studies of all antibiotic allergies appeared less likely to find an association between AMR and allergy status.

The clearest signals of an association between AMR and allergy came from studies of MRSA and penA. The vast majority of studies looking at MRSA in penA patients found a positive association. It is well known that penA labels drive antibiotic prescribing towards macrolide, tetracyclines, quinolones and cephalosporins^1,11^ and an association between quinolones and cephalosporins and MRSA colonization is also well described^46,48,51^ providing a plausible mechanism for this effect. However, an increased risk of MRSA was also associated with patients who had an allergy to any antibiotic,^15,33^ and it is highly likely that other factors apart from antibiotic exposure secondary to penA also mediate this risk, such as increased exposure to health care with an increased risk of cross-infection.

PenA-labelled patients in certain groups such as patients with CF or inflammatory bowel disease and those undergoing abdominal surgery had an increased risk of MRSA and therefore could be considered as populations to target for allergy assessment. One study found that cases of MRSA PJI were reduced in patients with penicillin or cephalosporin allergy,^44^ which may be attributable to the use of broader-spectrum vancomycin prophylaxis in these patients, raising questions about the appropriateness of prophylactic agents, more than arguing for penA de-labelling. An association between VRE and PenA was seen in hospital studies, but not in the general population, probably because the prevalence of VRE is so low in the latter. PenA is associated with increased cephalosporin prescriptions; therefore, these findings are not unexpected as exposure to cephalosporins has been linked to the isolation of VRE as well as resistant pneumococci and resistant gram-negative infections.^52^ A clear relationship between resistance in *S. pneumoniae* bloodstream infections and penA has not been established, and more data are needed.

Similarly, the association between antibiotic allergies and AMR in Gram-negative organisms is less clear with studies reporting conflicting results, however, compared with studies investigating MRSA, sample sizes in these studies tended to be much smaller and reported different AMR outcomes, so methodological issues may explain the mixed results. Both increased antibiotic use and ciprofloxacin use have been linked to the emergence of ESBL-producing bacteria.^53–55^ and might explain why ESBL acquisition may be associated with penA; however, this was not demonstrated in the included studies. Studies reporting on the association between ESBL infection/colonization all looked at different patient populations that likely had different antibiotic exposure. Additionally, studies with small sample sizes were more likely to report either no correlation or a negative correlation between ESBL infection/colonization and antibiotic allergy labels; no sample size calculations were provided, and lack of statistical power is highly likely to affect these studies.

The definition of MDR organisms varied from study to study highlighting the lack of consensus in defining MDR organisms. This inconsistency in reporting impedes comparison and meta-analysis of studies; thus, the true impact of penA on MDR is difficult to ascertain. Additionally, some studies reported on rates of colonization and other rates of infections, which also makes comparisons and meta-analyses difficult. In order to improve future studies investigating AMR, a consensus definition of MDR needs to be widely adopted and implemented to allow for a more accurate understanding of AMR.

Several studies have reported on the benefits of penA de-labelling.^56^ However, we found that only two studies^29,40^ looked at the impact of antibiotic allergy assessment and subsequent use of penicillins on AMR. One included participants with low-risk penicillin histories undergoing de-labelling of incorrect penA, while another included participants with high-risk penA undergoing de-sensitization. Trubiano et al.^29^ reviewed long-term outcomes post-allergy testing but did not find a reduction in MDR organism isolation in patients who underwent antibiotic allergy testing. In Rodriquez-Alarcon et al.’s^40^ study, first-time MDR acquisition was only seen in the control group who had not undergone desensitization but this difference was not statistically significant. Small participant numbers may have affected the power of both these studies, and additional studies are needed. Future studies should ideally include multicentre RCTs comparing penA de-labelling with standard of care with longitudinal measures of AMR (e.g. culture and resistome analysis). The studies in this review were largely based in secondary care, and as antibiotic use is highest in primary care,^57^ future research needs to include primary care patients.

All of the studies reviewed were observational in design and therefore limited by bias associated with observational studies, including confounding. In analyses of the association between penA and AMR, potential confounding factors are those that increase both the risk of AMR and penA. Several factors are associated with penA, such as age, sex, socioeconomic status (IMD), presence of co-morbidities and increased healthcare exposure.^1,50^ These factors are also risk factors for AMR and should therefore be included in multivariable analyses. While some of the studies in this review did attempt to account for confounding, they often did not include all the appropriate confounders. It is noteworthy that large studies that did account for appropriate confounders all confirmed an association between MRSA and penA. Another significant limitation is that patients in the assessed studies received various different antibiotics with each antibiotic applying different selective pressures, which might explain the differences seen in the presence of AMR. Exposure to antibiotics was not reported consistently; therefore, assessing the impact specific antibiotics had on AMR was not possible.

Small sample sizes in many of these studies may have limited their statistical power to detect clinically meaningful differences, in particular studies reporting on resistance in Gram-negative bacteria. The addition of published conference abstracts in this review allowed for further understanding to be gained and potentially reduced the impact of publication bias; however, this is limited by the inability to sufficiently review study methodology.^58^

## Conclusions

PenA labels are associated with AMR, in particular MRSA and VRE, but a link between penA and resistance in Gram-negative bacteria is less clear. This association between penA and AMR is likely to be due, at least in part, to differences in antibiotic exposure between patients labelled as penA allergic and those who are not. De-labelling penA labels is therefore a potentially important means of reducing AMR by reducing exposure to antibiotics that drive AMR. While we have found an association between penA and AMR and a plausible mechanism, we cannot establish causality due to the observational nature of current studies. We also found methodological issues that may interfere with our ability to detect associations between different antibiotic allergies and AMR.

Further research is therefore warranted to investigate the impact of penA and other specific antibiotic allergies on AMR, including RCTs that measure the impact of de-labelling on subsequent antibiotic use and AMR.

## Supplementary Material

dlaf002_Supplementary_Data

## Transparency declarations

S.A. has no conflicts of interest to declare. J.S. is a member of the British Society for Allergy and Clinical Immunology (BSAC) penA working party and the European Society for Clinical Microbiology and Infectious Diseases guideline for antibiotic allergy, co-led the BSAC massive open access online (MOOC) penA course, and has received funding from the Engineering and Physical Sciences Council, National Institute for Health and Care Research (PGfR, HS&DR, i4i, HTA), Welcome Trust and Medical Research Council.

## Supplementary data

Table S1 is available as Supplementary data at *JAC-AMR* Online.
