# Supplementary material for: A rapid literature review of the impact of penicillin allergy on antibiotic resistance
Source: JAC Antimicrob Resist. 2025 Jan 21;7(1):dlaf002. doi: 10.1093/jacamr/dlaf002 (PMC11747217; doi:10.1093/jacamr/dlaf002)
Supplement: dlaf002_Supplementary_Data [file dlaf002_supplementary_data.docx]

**Supplementary Material**

Table S1. Study population, setting, design, size and exposure for included studies

| **Authors** | **Publication Year** | **Population/setting** | **Design** | **Size** |
| --- | --- | --- | --- | --- |
| Neuman et al^34^ | 2007 | Patients with *Streptococcus pneumoniae* bacteraemia (34 sites, US) | Retrospective cohort study | 1574 patients |
| Reddy et al*^15^ | 2013 | Prevalence of PenA was compared in a cohort of inpatients with VRE, MRSA and *C. difficile* and the general hospital population (single centre, US) | Retrospective review of EHR | VRE 426 patients, MRSA 4438 patients  General hospital population - 369721 patients |
| Macy et al^39^ | 2014 | Adult inpatients (single centre, US) | Retrospective matched case-control (1:2) study using EHR | 51 582 cases |
| Reddy et al*^16^ | 2014 | Inpatients who received vancomycin +/- had VRE infection (single centre, US) | Retrospective review of EHR | 6,720 patients with penA and 89,352 without penA |
| Knezevic et al*^17^ | 2015 | Inpatients in a tertiary hospital (single centre, Australia) | Retrospective cross-sectional study using data from the 2013 and 2014 National Antimicrobial Prescribing Surveys (NAPS) | 725 patients |
| Tan et al^44^ | 2016 | Adult patients post total joint arthroplasty (two centres, US) | Retrospective cohort study | 10 391 patients (2657 with exposure) |
| Khumra et al^32^ | 2017 | Adult, liver transplant patients (single centre, Australia) | Retrospective matched-cohort study | 313 patients (51 cases) |
| Jones et al*^18^ | 2018 | Patients with cystic fibrosis (single centre, US) | Retrospective cohort study | 271 patients |
| Sousa-Pinto et al^35^ | 2018 | Adult inpatients (multicentre, Portugal) | Retrospective cohort study using EHR | 102 872 with exposure |
| Dewart et al^38^ | 2018 | Adult inpatients patients with urinary tract infections (single centre, US) | Retrospective cohort study using EHR | 6361 patients (1252 with exposure) |
| Blumenthal et al^36^ | 2018 | Primary care patients (multicentre, UK) | Population based matched cohort study using data from The Health Improvement Network (THIN) | 301 399 (61 141 cases) |
| Galant-Swafford et al*^20^ | 2018 | Inpatient (single centre, US) | Retrospective case-control study using EPIC electronic medical record database | Not specified |
| West et al^1^ | 2019 | Primary care patients (multicentre, UK) | Exact matched cohort study using general practice electronic health records (ResearchOne) | 130 571 patients matched with 1 892 835 controls |
| Strazzulla et al^27^ | 2020 | Adult ICU inpatients (single centre, France) | Retrospective cohort study | 1174 patients (41 with exposure) |
| Motoa et al^25^ | 2020 | Adult liver transplant recipients (single centre, US) | Retrospective cohort study | 174 contols:62 cases |
| Lam et al^33^ | 2020 | Adult inpatients screened for MRSA and VRE (single centre, US) | Retrospective cohort study | 1053 patients screened for MRSA (333 with exposure) and 290 screened for VRE (126 with exposure) |
| Trubiano et al^29^ | 2020 | Adult patients who underwent antibiotic allergy testing with ≥ 1 inpatient admission were matched with controls who did not undergo allergy testing (single centre, Australia) | Matched 1:1 case control study | 310 patients (155/arm) |
| Baxter et al^3^ | 2020 | Adult inpatients (single centre, UK) | Point prevalence study | 583 patients (80 with exposure) |
| Schlosser et al^45^ | 2020 | Adult patients undergoing open ventral hernia repair (single centre, US) | Retrospective cohort study using prospective institutional database undergoing open ventral hernia repair | 1178 |
| Leone et al^24^ | 2021 | Adult intensive care unit inpatients (multicentre, France) | Retrospective cohort study | 7146 (440 with exposure) |
| Nelson et al*^42^ | 2021 | Organ transplant patients admitted with a primary infectious process (single centre, US) | Retrospective cohort study using data from data from the National Inpatient Sample database | 50 069 patients (1170 with exposure) |
| Ahmed et al*^19^ | 2022 | Comparison of patient isolates in cohorts of patients isolating *S. pneumoniae* in blood culture, *S. aureus* in blood cultures, *S. pneumoniae* in sputum culture and *H. influenzae* in sputum culture (single centre, UK) | Observational study, Service evaluation | 297 patients *S. penumoniae* in blood culture, 783 patients with *S. aureus* in blood cultures, 156 patients with *S. pneumoniae* in sputum culture and 719 patients with *H. influenzae* in sputum culture |
| Greenwald et al*^22^ | 2022 | Patients CD and UC (multi-centre, US) | Retrospective cohort study | 8699 patients with CD, 7536 patients with UC |
| Chakravorty et al^31^ | 2022 | Adult inpatients reviewed on prospective audit and feedback rounds (single centre, Australia) | Retrospective cohort study | 630 patients (103 with exposure) |
| Naciri et al^26^ | 2022 | Adult inpatients with intra-abdominal infections (single centre, France) | Retrospective 1:1 case-control study | 86 patients |
| Strazzulla et al^28^ | 2022 | Adult ICU inpatients (single centre, France) | Retrospective cohort study | 3332 patients (132 with exposure) |
| Zhang et al^30^ | 2022 | Adult inpatients undergoing solid-organ transplants (single centre, US) | Retrospective cohort study | 1700 (285 with exposure) |
| Brennan et al*^23^ | 2023 | Patients who had received a LVAD followed up for 5 years post -insertion | Retrospective study using data from the TriNetX Research Network | 440 patients (60 with exposure) |
| Greenwald et al*^21^ | 2023 | Patients with UC including those receiving 5-aminosalicylate (5-ASA) and anti-TNF agents (multi-centre, US) | Retrospective matched cohort study | 8,612 patients with UC |
| Rodríguez-Alarcón et al^40^ | 2023 | Adult penA inpatients with infection who underwent antibiotic desensitization matched with PenA controls treated with alternative antibiotics (single centre, Spain) | Retrospective 3:1 case-control study | 56 patients |
| Chadha et al^37^ | 2023 | Adult inpatients with complicated odontogenic infections (single centre, US) | Retrospective cohort study | 150 patients (26 with exposure) |
| Jones et al^41^ | 2024 | Patients undergoing gastrointestinal surgery (single centre, UK) | Retrospective cohort study using EHR and national SSI surveillance data | 3644 patients (461 with exposure) |
| Stevoska et al^43^ | 2023 | Adult patients post total Hip or Knee arthroplasty (single centre, Austria) | Retrospective cohort study | 3419 patients with hip arthroplasty (exposure in 206) and 2666 patients with knee arthroplasty (211 with exposure) |
| *conference abstract only CD - Crohn’s disease EHR – Electronic health record, LVAD - Left ventricular assist devices, UC - ulcerative colitis, US – United States, UK – United Kingdom, UTI – Urinary tract Infection | | | | |
